# Use of Multifunctional Nanoclay@VS_2_ Nanoflowers in the Adsorption and Photocatalytic-Based Removal of Drug Molecules and Azo Dyes

**DOI:** 10.1021/acsomega.5c12735

**Published:** 2026-06-02

**Authors:** Fulya Sütçü Güney, Oktay Özkan, İbrahim Narin, Erkan Yılmaz

**Affiliations:** 1 Faculty of Engineering, Institute of Science and Technology, 52958 Erciyes University,Kayseri, Talas 38280 ,Turkey; 2 Environmental Engineering Department, 52958 Erciyes University, Kayseri, Talas 38280, Turkey; 3 Department of Analytical Chemistry, Faculty of Pharmacy, Erciyes University, Kayseri, Talas 38280, Turkey; 4 Technology Research & Application Center (TAUM), 52958 Erciyes University, Kayseri 38039, Turkey; 5 ERNAM-Nanotechnology Research and Application Center, 52958 Erciyes University, Kayseri 38039,Turkey; 6 Erciyes Teknopark-ChemicaMed Chemical Inc., Erciyes University Technology Development Zone, Kayseri 38039, Turkey

## Abstract

The scarcity of clean water resources has become a critical global environmental issue due to rapid population growth and unplanned industrialization, which have significantly disrupted natural purification processes. Moreover, the increasing coexistence of persistent organic dyes and pharmaceutical pollutants in wastewater, which are resistant to conventional treatment methods, poses a serious threat to aquatic ecosystems and human health. This situation highlights the urgent need for the development of efficient, sustainable, and multifunctional treatment strategies capable of addressing diverse classes of contaminants simultaneously. The main objective of this study is to develop a multifunctional nanocomposite material capable of integrating adsorption and photocatalytic processes for the effective removal of both dye and pharmaceutical pollutants. In this context, the removal of methylene blue (MB), a widely encountered industrial dye, and escitalopram (ESC), a pharmaceutical contaminant, was investigated using multifunctional Nanoclay@VS_2_ nanoflowers. The synthesized Nanoclay@VS_2_ nanocomposite, which combines the high adsorption capacity of nanoclay with the photocatalytic activity of VS_2_ nanoparticles, was evaluated as a dual-functional treatment material. The results demonstrated that the optimum conditions for the adsorption of MB were pH 6.0, 15 mg of adsorbent dosage, and 10 min of contact time, achieving removal efficiencies in the range of 93–99%. Photocatalytic degradation of MB under UV irradiation exceeded 95% within 270–480 min, with optimum conditions of pH 4.0 and 200 mg photocatalyst. Reusability studies confirmed that the material maintained its performance for at least three cycles. For ESC removal, adsorption experiments achieved an efficiency of 87% under similar conditions (pH 6.0, 15 mg), while photocatalytic degradation under UV light reached 96% within 330 min at pH 2.0 using 100 mg of photocatalyst. Overall, these findings demonstrate that Nanoclay@VS_2_ nanoflowers exhibit significant potential as efficient, sustainable, and multifunctional materials for the removal of structurally different organic pollutants from wastewater.

## Graphical abstract

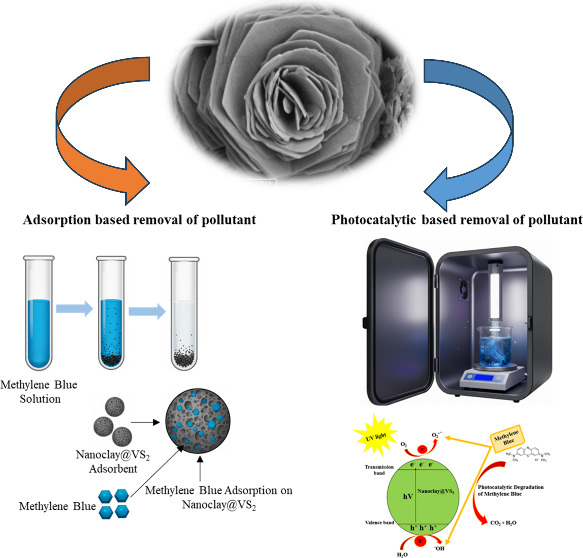

## 1 Introduction

Although approximately two-thirds of the Earth’s surface is covered with water, less than 1% of existing water resources are directly suitable for human consumption. Human activities such as uncontrolled population growth, rapid urbanisation and unplanned industrialization have led to a decline in both the quantity and quality of existing water resources.^1,2^ This situation has led to the disruption of natural purification processes and a gradual decrease in potable water resources. As a result, drinking water has become a limited and competitive resource in many regions.^3,4^ Therefore, water scarcity is now one of the fundamental environmental problems that need to be solved, especially in developing countries.^5,6^ Water pollution is one of the most significant environmental problems encountered worldwide. Efforts have intensified to develop environmentally friendly and economical treatment methods to protect clean water sources and prevent the uncontrolled discharge of highly polluted wastewater into receiving environments.^7^ Although many methods based on physical, chemical, and biological principles are used in wastewater treatment, increasing water demand, strict health standards, and emerging new-generation pollutants limit the effectiveness of these classical methods.^3^ At this point, nanomaterials stand out as noteworthy alternatives in water treatment and water quality management due to their unique physicochemical properties, high efficiency, economic advantages, and environmentally compatible structures.^8^ Nanomaterials offer effective solutions for both the detection and removal of pollutants due to their small size, large surface area, high adsorption capacity, and photoelectronic and photocatalytic properties.^9,10^

Clay-based nanomaterials are widely utilized in water treatment technologies due to their high surface area, layered crystalline structure, ion-exchange capacity, and chemical stability. Specifically, nanoclay structures such as montmorillonite and kaolinite have been reported as effective adsorbents for removing organic dyes, heavy metals, and pharmaceutical pollutants from aqueous media.^11,12^ The layered structure and surface hydroxyl groups of these materials provide numerous active adsorption sites for sequestration processes.^13^ In recent years, semiconductor materials belonging to the transition metal dichalcogenides (TMDs) class, such as VS_2_, have been investigated as promising candidates for photocatalytic water treatment applications. Due to its layered crystal structure, high electrical conductivity, and suitable bandgap, VS_2_ can facilitate the oxidative degradation of organic pollutants by generating electron–hole pairs under light irradiation.^14,15^ Although nanoclay and VS_2_ materials have individually shown successful results in pollutant removal, studies on hybrid nanocomposite structures incorporating both materials remain quite limited. Integrating nanoclay with VS_2_ nanostructures can enhance pollutant removal performance by combining the high adsorption capacity of the nanoclay with the photocatalytic activity of VS_2_.^16^

The use of nanomaterials provides significant advantages, particularly in the removal of pollutants that are difficult to eliminate using conventional methods, such as organic dyes. These dyes, widely used in industrial processes, are among the serious pollutants of water resources and can cause long-term damage to the environment. Furthermore, the presence of pharmaceutical active ingredients (PAIs) in aquatic environments has emerged as a critical environmental concern. Among these, Escitalopram (ESC), a common antidepressant, is frequently detected in wastewater due to its high consumption and resistance to traditional biological treatment processes.^17^ Therefore, evaluating the degradation of both dyes and pharmaceuticals is essential for developing versatile water treatment technologies. In this context, nanomaterials are considered a promising environmentally friendly approach for the adsorption and photocatalytic degradation of organic pollutants.^18^ The adsorption method is an effective technique for removing both organic and inorganic pollutants due to its simple design, low cost, high efficiency, reusability, and environmentally friendly structure. Furthermore, its ability to effectively remove even pollutants at very low concentrations is one of the key advantages that sets this method apart.^19^ Another effective method that combines energy conversion and degradation in the removal of pollutants is photocatalytic degradation. Photocatalysis is based on the principle of decomposing or converting chemical compounds using light energy. In this process, semiconductor photocatalysts absorb light and produce active radicals; these radicals remove pollutants through oxidation or reduction.^20,21^

Therefore, the novelty of this study lies in the synthesis of a novel Nanoclay@VS_2_ hybrid nanocomposite with a nanoflower morphology and the investigation of its performance in the removal of MB dye and ESC through both adsorption and photocatalytic processes.

In this study, Nanoclay@VS_2_ NFs material was preferred as a photocatalyst due to its strong oxidative and adsorptive properties. VS_2_ has a wide light absorption capacity thanks to its narrow bandgap and exhibits high photocatalytic activity under UV light. On the other hand, the nanoclay component facilitates the retention of pollutants on the surface due to its large surface area and high adsorption capacity, thereby increasing the photocatalytic reaction rate.^22^ Within this scope, the Nanoclay@VS_2_ NFs material synthesized in the study was utilized in the removal of MB and ESC from aqueous solutions through both adsorption and photocatalytic degradation mechanisms. The results obtained demonstrate that these methods provide high efficiency in pollutant removal and that environmentally friendly nanomaterials can be considered an effective alternative in wastewater treatment.

## 2 Experimental Section

### 2.1 Chemicals and Reagents

In the present study, Nanoclay (with a particle size of 30 nm) was obtained ftom Nanografi Nano Technology. Thioacetamide (Merck) and ammonium monovanadate (Merck) was used for the synthesis of VS_2_ nanoparticles. Methylene blue (MB) dye, used as a model organic pollutant, and escitalopram (ESC), a pharmaceutical organic pollutant, were obtained from Sigma-Aldrich. The following reagents were used in the experiment: ammonium solution (25%), ammonium chloride (NH_4_Cl), sodium dihydrogen phosphate (NaH_2_PO_4_), and sodium hydrogen phosphate (Na_2_HPO_4_) obtained from Merck. Hydrochloric acid (HCl) from Isolab, orthophosphoric acid (H_3_PO_4_) 85% from Carlo Erba and NaOH from Sigma-Aldrich was supplied. Deionized water (resistance 18.2 MΩ·cm) was obtained using a Milli-Q deionized water system (Millipore, USA).

### 2.2 Instrumentation

FE-SEM and EDX analyses were conducted on a ZEISS GEMINI 500 model with a magnification ratio of 50.000 in order to investigate the structure, morphology, and chemical composition of Nanoclay@VS_2_ NFs. The analysis of the crystal structure was conducted utilizing a Panalytical Empyrean X-ray powder diffractometer (XRD). The band structures of the materials were elucidated by means of Fourier Transform Infrared Spectroscopy (FT-IR) analysis, which was performed using a PerkinElmer 400 FT-IR/FT-IR Spectrometer Spotlight 400 Imaging System. The PerkinElmer Lambda 25 UV–vis spectrophotometer was utilized to conduct the MB dye analysis. Escitalopram analysis was performed using an Agilent 1260 Infinity II model UPLC (800 bar limited) device.

### 2.3 Synthesis of Nanoclay@VS_2_ Nanoflowers

The synthesis of Nanoclay@VS_2_ NFs was initiated by weighing 50 mg of nanoclay in a 100 mL beaker and subsequently adding 10 mL of pure water. The substance was then exposed to ultrasonic vibration for a period of 20 min, with the objective of achieving complete distribution of nanoparticles. Subsequently, 3.33 g of thioacetamide (44.31 mmol) was mixed in 12 mL of distilled water in a second 100 mL beaker and subjected to mixing at a temperature of 70–80 °C using a magnetic stirrer to ensure dissolution. Subsequent to dissolution, stirring was continued at the same temperature. A quantity of 649.88 mg of ammonium monovanadate (5.56 mmol) was added to 30 mL of distilled water and 8 mL of ammonium solution in a third 100 mL beaker. The contents were stirred using a magnetic stirring until the material dissolved. Subsequent to the dissolution of the contents of the second and third beakers, the second beaker’s solution was gradually introduced to the third beaker, with the mixers continuing to operate. Following the stirring of the newly obtained solution in a magnetic stirrer for a period of 15–20 min, the 50 mg nanoclay–water mix in the first beaker was placed in the magnetic stirrer and the new solution was added dropwise. The last solution was achieved through the put together of all constituent materials, which were subsequently subjected to magnetic stirring for a duration of 15–20 min. The solution obtained in the final stage was transferred to a Teflon hydrothermal synthesis vessel and processed in a hydrothermal autoclave reactor at 180 °C for 24 h. The Nanoclay@VS_2_ NFs were then collected using a centrifuge and thoroughly washed with pure water. The Nanoclay@VS_2_ NFs were subjected to a drying process at a temperature of 100 °C for a duration of 1 h. Subsequent to this, the material was reduced to the smallest possible particle size by means of an agate mortar.

### 2.4 Adsorption-Based Removal Method for MB and ESC

Optimization studies of the adsorption method were carried out and optimum conditions were determined. For optimization of the method, a 10 mL solution containing 1.5 mL of pH 6.0 phosphate buffer was prepared in a falcon tube with an MB concentration of 10 mg·L^–1^. Fifteen mg of Nanoclay@VS_2_ NFs was added to this solution. The mixture was kept in an ultrasonic bath for 10 min to adsorb the MB in the solution onto the Nanoclay@VS_2_ NFs. Then, Nanoclay@VS_2_ NF particles were subjected to shaking for 10 min and ultrasonic bath for 5 min. Then, Nanoclay@VS_2_ NFs were isolated from the solution by centrifuging at 4000 rpm for 5 min. Approximately 1.5–2 mL of the liquid phase remaining on the Nanoclay@VS_2_ NFs that settled to the bottom after the centrifugation process was taken and prepared for measurement in a UV–vis spectrophotometer at a wavelength of 664 nm. The adsorption method and UV–vis spectrophotometer measurement procedure are shown in Figure S1. For the optimization of the ESC adsorption process, 15 mg of Nanoclay@VS_2_ NFs were added into a falcon tube containing 1.5 mL of pH buffer and a 10 mg·L^–1^ ESC solution, reaching a final volume of 10 mL. The mixture was subjected to 10 min of mechanical shaking by using a shaker device. Subsequently, the Nanoclay@VS_2_ NFs were separated from the aqueous phase by centrifugation at 4000 rpm for 2 min. In the final step, the supernatant was collected via a syringe, filtered through a 0.22 μm membrane filter, and then analyzed using a UPLC-DAD system. The adsorption percentage was calculated using the following eq 1.

$$\mathrm{Adsorption}\;(\%)=\left(\frac{C_{0}-C_{t}}{C_{0}}\right)\times 100$$ 1

Here, C_0_ (mg·L^–1^) represents the initial concentration of MB and ESC, and C_t_ (mg·L^–1^) represents the concentration of analytes at time t.

The adsorption capacity of Nanoclay@VS_2_ NFs was calculated using eq 2 below.^23^

$$Q_{e=}\frac{C_{0-C_{e}}}{m}\times V$$ 2

In this equation, Q_e_ (mg·g^–1^) indicates the equilibrium adsorption capacity, C_0_ (mg·L^–1^) and C_e_ (mg·L^–1^) represent the initial and equilibrium concentrations of MB, respectively, V (L) is the volume of the MB solution, and m (g) is the mass of the adsorbent.^24^

### 2.5 Photocatalytic Degradation-Based Removal Method for MB and ESC

The photocatalytic performance of Nanoclay@VS_2_ NFs were investigated in a 150 mL model solution containing a specific MB concentration. In the study, 100 mg of Nanoclay@VS_2_ NFs were added to 150 mL of solution, and the mixture was stirred with a magnetic stirrer in a dark environment that did not allow light to pass through for 12 h to ensure complete adsorption of MB. Following the stirring process, the mixture was transferred to the photocatalytic reactor and exposed to light using a 400 W UV-halogen lamp placed at the center of the reactor. In order to monitor the photocatalytic degradation rate, 2.0 mL samples were taken at specific time intervals and analyzed using a UV–vis spectrophotometer. The photocatalytic degradation process of MB is shown in Figure S2. The photocatalytic performance of Nanoclay@VS_2_ NFs for ESC removal was also investigated using a 150 mL model solution containing a specific concentration of ESC at various pH values. In this procedure, 100 mg of Nanoclay@VS_2_ NFs were added to the solution. To ensure adsorption–desorption equilibrium, the mixture was stirred with a magnetic stirrer for a specific period in a dark environment. Subsequently, the suspension was transferred to the photocatalytic reactor and irradiated using a 400 W UV-halogen lamp. To monitor the degradation progress, 2.0 mL samples were collected at predetermined time intervals, filtered through a 0.22 μm membrane filter, and analyzed using the UPLC-DAD system. The photocatalytic removal efficiency was calculated using the following equation:

$$\mathrm{Degradation}\;(\%)=\left(\frac{C_{0-C_{t}}}{C_{0}}\right)\times 100$$ 3

Here, C_0_ (mg·L^–1^) represents the sample taken before the model solution is placed in the photocatalytic reactor, while C_t_ (mg·L^–1^) represents the sample taken after exposure to the UV lamp for t (minutes). The rate and kinetics of MB degradation were investigated using pseudozero-order, pseudo-first-order, and pseudo-second-order kinetic models (eqs 4–6).

$$\mathrm{Pseudozero}\;\mathrm{degree},Ct=k_{o}t+C_{0}$$ 4

$$\mathrm{Pseudo}\;\mathrm{first}\;\mathrm{degree},In\left(\frac{Co}{Ct}\right)=k_{1}t$$ 5

$$\mathrm{Pseudo}\;\mathrm{second}\;\mathrm{degree},\frac{1}{C_{t}}=\frac{1}{C_{0}}+k_{2}t$$ 6

Here, C_0_ is the initial MB concentration (mg·L^–1^), C_t_ is the MB concentration at time t (mg·L^–1^), t is the contact time (hours) and *k*_0_, *k*_1_ and *k*_2_ are the zero, first and second-order rate constants, respectively.^24^

## 3 Results and Discussion

### 3.1 Characterization of Nanoclay@VS_2_ NFs

A 5000 times magnification and 2 μm scale FE-SEM images of Nanoclay and VS_2_ NP is shown in Figure 1A,B. The FE-SEM image of Nanoclay shows that the material has a distinctly round morphology. It is thought that the round morphology may have been formed depending on the synthesis method of Nanoclay and the process parameters used. The rough surface structure and the fact that it is covered with fine particles contribute to nanoclay high surface area and adsorption capacity (Figure 1A). VS_2_ NP has been observed to have two-dimensional layer or flake-like structures^25,26^ and morphological features composed of ‘flower-like’ nanostructures^27^ (Figure 1B). The FE-SEM image of the synthesized Nanoclay@VS_2_ NFs at 10.000 magnification and 1 μm scale is shown in Figure 1C. It was observed that the nanoclay particles were distributed homogeneously and in small structures on the surface of the VS_2_ layers. It is considered that the nanocomposite material synthesized by combining nanoclay with VS_2_ nanoparticles could potentially provide high efficiency in adsorption and photocatalytic applications. The EDX analysis spectrum for Nanoclay@VS_2_ NFs showed peaks corresponding to V, S, C, O, Al, Si, Ca, Fe, and Zn atoms (Figure 1D). The EDX analysis was performed by scanning a wide area representing the nanocomposite. FE-SEM and EDX results confirmed that Nanoclay@VS_2_ NFs was successfully synthesized.

**Figure 1:**
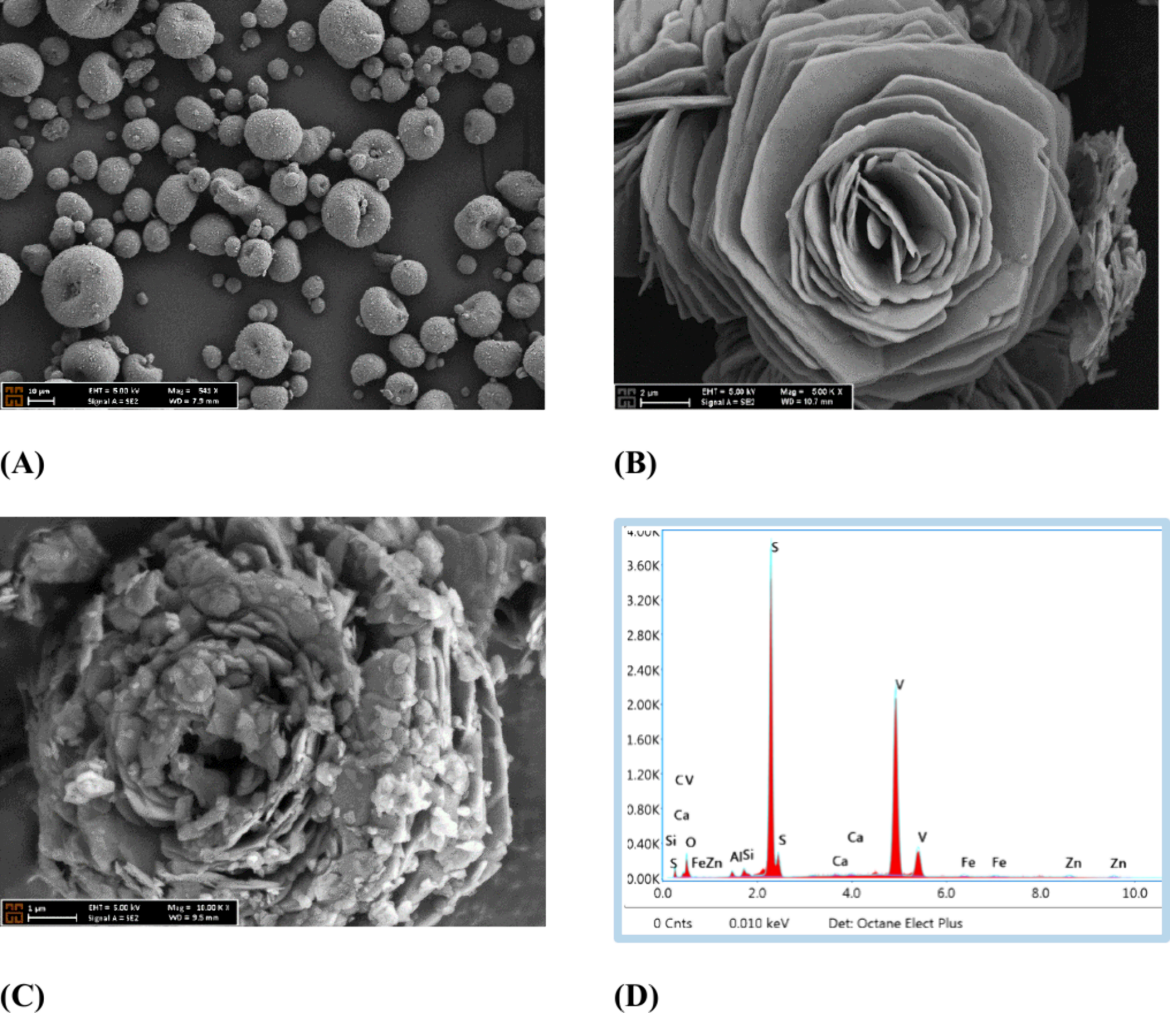
(A) FE-SEM images of nanoclay, (B) VS_2_ NP, and (C) Nanoclay@VS_2_ NFs, and (D) EDX spectrum of Nanoclay@VS_2_ NFs.

The XRD spectra of Nanoclay, VS_2_ NP, and Nanoclay@VS_2_ NFs are shown in Figure 2A. VS_2_ exhibits typical diffraction peaks at 2θ = 15.38°, 35.74°, 45.23°, 57.14°, 58.33°, 59.56°, 69.26°, and 75.72°, indicating a hexagonal layered structure, corresponding to the (001), (011), (012), (110), (103), (111), (201), and (202) planes, respectively.^25,28^ The (001) Miller index defines the layered stacking of the S–V–S layer, which is critical for examining the structure of VS_2_. The characteristic (001) diffraction at 15.4° indicates a stacked lamellar structure.^29^ The intense and sharp (001) diffraction peak at 2θ = 15.38° indicates that the layered structure of VS_2_ has a high-quality and regular layered stacking.^26^ Narrow and sharp peaks indicate the high crystallinity of the synthesized nanoparticle. No peaks originating from impurities were detected, indicating the formation of pure VS_2_ nanoparticles.^25^ In the XRD spectrum of Nanoclay, the diffraction peaks observed at 2θ = 12.28°, 24.78° and 62.26° correspond to the (001), (002), and (1–52) planes, respectively.^30,31^ The (001) and (002) peaks observed in the XRD spectrum of nanoclay confirm the layered structure of the material, while the high-angle peaks (1–52) indicate that it has a regular and dense crystalline structure. When compared with the literature, the XRD data of nanoclay is consistent with kaolinite. The 2θ = 12.28°, 15.38°, 24.78°, 35.74°, 45.23°, 57.14°, 58.33°, and 59.56° in the XRD spectrum of Nanoclay@VS_2_ NFs indicate various planes of the material’s crystalline structure. These diffraction peaks represent Nanoclay@VS_2_ NFs. These diffraction peaks prove that Nanoclay and VS_2_ are combined in a homogeneous structure and that the nanocomposite maintains a high degree of crystalline order. When compared with other studies in the literature, the characteristic peaks of the Nanoclay@VS_2_ NFs were found to be consistent with the diffraction peaks.

**Figure 2:**
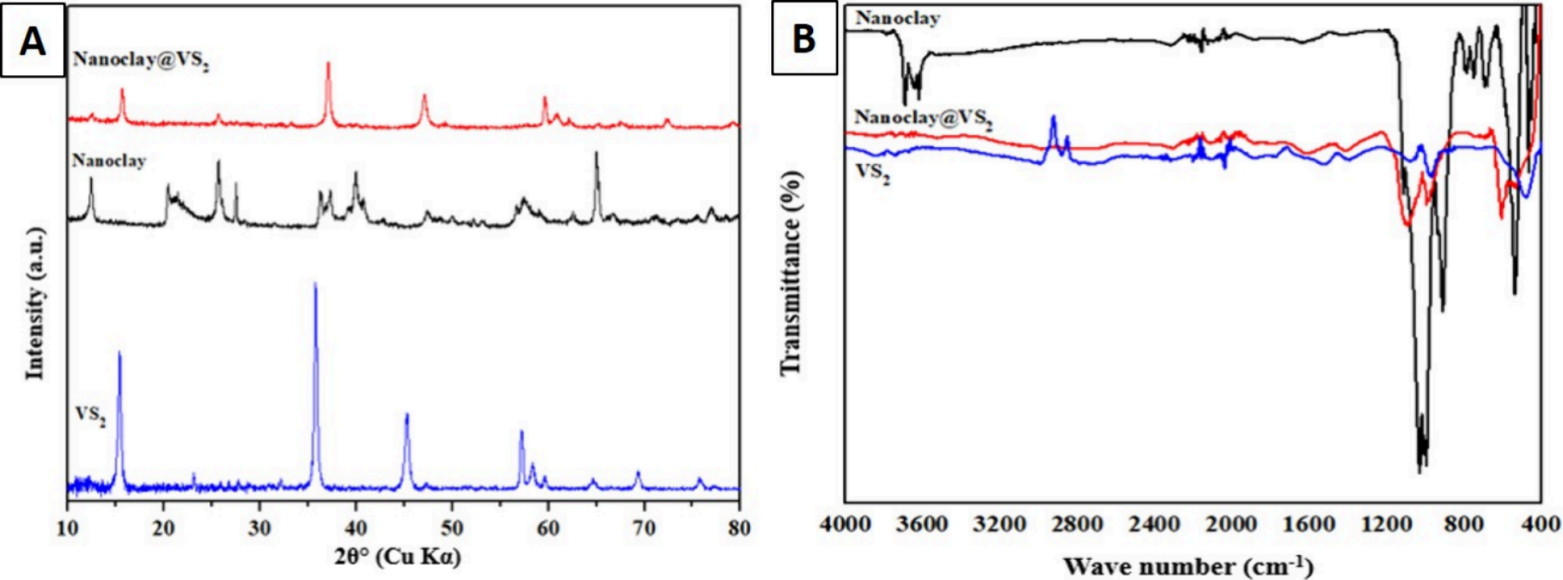
(A) XRD spectra of VS_2_ NPs, nanoclay, and Nanoclay@VS_2_ NFs; (B) FT-IR spectra of nanoclay, VS_2_ NPs, and Nanoclay@VS_2_ NFs.

The peaks in the FT-IR spectrum of Nanoclay@VS_2_ NFs reflect the vibrations that indicate the chemical structure of the material and the bonds it contains. The FT-IR spectra of Nanoclay, VS_2_ NP, and Nanoclay@VS_2_ NFs measured in the range of 400 to 4000 cm^–1^ are shown in Figure 2B. In the FT-IR spectrum of Nanoclay, the peaks observed at 3692.3 cm^–1^, 3648.4 cm^–1^, and 3617.6 cm^–1^ were determined to be associated with O–H octahedral stretching vibrations characteristic of kaolinite-like clay minerals. The strong bands at 1114.5 cm^–1^, 1030.7 cm^–1^, and 1007.1 cm^–1^ are attributed to Si–O stretching vibrations in the structure. The peak at 910.82 cm^–1^ is thought to correspond to Al–Al–OH stretching. The peak at 794.52 cm^–1^ indicates Al–Mg–OH stretching, while the peak at 752.88 cm^–1^ indicates Si–O–Al stress. The peak at 693.7 cm-1 was found to represent stress vibrations associated with Si–O and Si–O–Al bonds. The 538.23 cm^–1^ peak is associated with metal–oxygen (Si–O or Al–O) vibrations originating from Si–O–Al and Si–O vibrations, while the 465.49 cm^–1^ peak is associated with Si–O vibrations.^31,33,35^ These peaks are consistent with the bond vibrations characteristic of clay minerals such as kaolinite, indicating that the crystalline structure of the nanoclay material is well preserved. Surface hydroxyl groups or functional groups that may arise from the interaction between the clay and VS_2_ in the nanoclay content may give signals in this region. The peaks at 999.67 cm^–1^ and 1033.5 cm^–1^ were found to reflect Si–O vibrations specific to the kaolinite structure.^32^ The peaks observed in the range of 461.03 to 601.66 cm^–1^ are attributed to the regular structure of the S–V–S layers and metal–sulfur vibrations.^33,34^ A large portion of the obtained peaks are consistent with characteristic bond vibrations indicating the presence of both kaolinite and VS_2_. These findings demonstrate that Nanoclay@VS_2_ NFs was successfully synthesized and that the functional groups of its components were preserved in the structure, as revealed by FT-IR analysis.

### 3.2 Optical Study of Nanoclay@VS2 NFs

The optical properties (i.e., the absorption spectra and band gap) of Nanoclay@VS_2_ NFs were investigated using UV–vis spectroscopy, which involved measuring the light absorption of the sample over a range of wavelengths between 200 and 1100 nm (Figure 3). The absorption spectrum of Nanoclay@VS_2_ NFs revealed a maximum absorption peak at 382.08 nm. The observed photocatalytic activity is primarily governed by the electronic transitions from the occupied S 3p states in the valence band (VB) to the unoccupied V 3d states in the conduction band (CB). This transition corresponds to the excitation of electrons within the V–S coordination bonds of the hexagonal VS_2_ lattice. Specifically, the narrow bandgap of VS_2_ facilitates a Ligand to Metal Charge Transfer (LMCT), where visible light provides sufficient energy to promote electrons from the sülfür dominated VB to the vanadium-rich CB, thereby generating the electron–hole (e^–^/h^+^) pairs essential for the degradation of MB and ESC.^36^ Furthermore, the Tauc plot, utilized for the calculation of direct band gaps, demonstrated that Nanoclay@VS_2_ NFs possesses a direct band gap of 3.77 eV (Figure 3).

**Figure 3:**
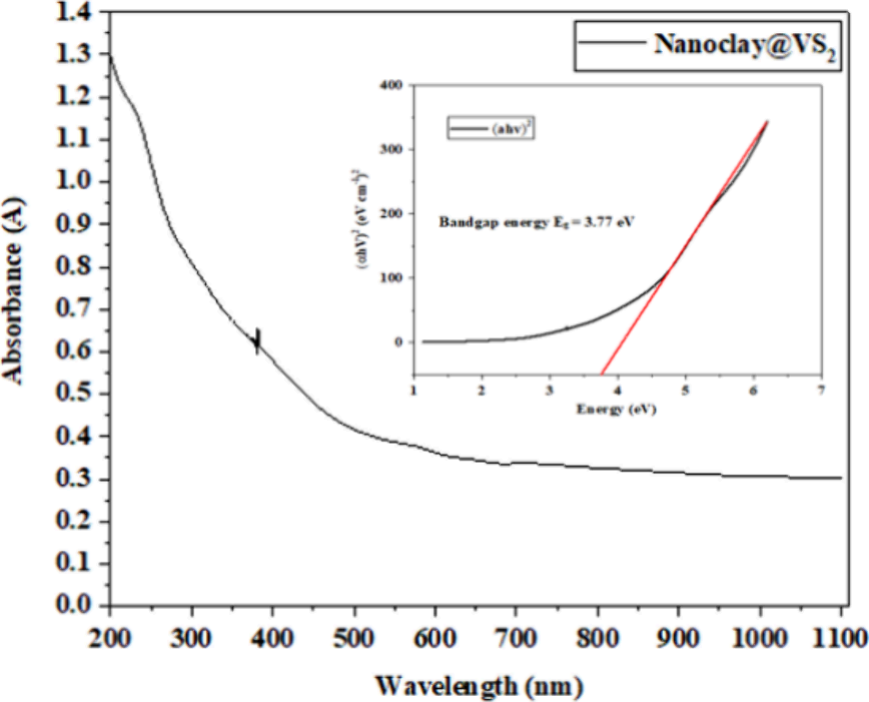
UV–vis spectrum and Tauc graph of Nanoclay@VS_2_ NFs.

The observed expansion in the optical band gap of the Nanoclay@VS_2_ nanocomposite is attributed to the quantum size effect and the synergistic interaction between the nanomaterial and the support matrix. The kaolinite used as a support is a wide-bandgap insulator (∼4.5–5.0 eV) that does not absorb visible light, thus serving primarily as a structural framework rather than a direct photocatalyst.^37^ The significant blue shift in the band gap energy is driven by the quantum confinement effect; the reduction of VS_2_ nanosheets and nanoflower petals to the nanoscale restricts charge carrier movement and discretizes energy states.^38,39^ Furthermore, the kaolinite matrix effectively suppresses the restacking and agglomeration of VS_2_ layers by acting as a physical spacer. This preserves the unique electronic and optical properties of the few-layered VS_2_ and prevents its transition toward bulk-like behavior.^40^ Consequently, the calculated E_g_ values confirm the successful engineering of the Nanoclay@VS_2_ architecture and its optimized semiconducting nature.

The synthesized Nanoclay@VS_2_ nanoflowers exhibited a direct band gap of 3.77 eV, representing a pronounced blue shift compared to the reported values for bulk VS_2_ (∼1.2 eV). This expansion is primarily attributed to the strong quantum confinement effect resulting from the growth of ultrathin VS_2_ nanosheets on the layered silicate templates of the nanoclay.^38,41^ The nanoclay matrix serves as a solid-state diluent that prevents electronic coupling and restacking of individual VS_2_ units, thereby preserving the high-energy transitions associated with two-dimensional nanostructures. Furthermore, the interfacial interactions between the transition metal sulfide and the oxygen-rich surface of the clay may induce a redistribution of the electronic density of states, further contributing to the widened optical band gap.^42^

### 3.3 Adsorption Studies of MB and ESC Using Nanoclay@VS2 NFs

#### 3.3.1 Calibration and Quantification of MB and ESC for Adsorption Experiments

A comprehensive investigation was conducted into the pivotal parameters influencing the adsorption of MB on Nanoclay@VS_2_ NFs, encompassing parameters such as pH of sample solution, adsorbent amount, adsorption time, temperature and adsorption capacity. The objective of this investigation was to optimize these parameters.

MB, a chemical compound of significant industrial and commercial importance due to its wide range of applications as an organic dye, was selected as the analyte. In the initial phase, a preliminary method calibration study was conducted for MB. For the purposes of this study, a stock MB solution was prepared at a concentration of 1000 mg·L^–1^. Concentrations were determined for the standard solution series, and the volumes to be taken from the standard solution were calculated. The measurements of the standard solution series were performed at a wavelength of 664 nm using a UV–vis spectrophotometer. Subsequently, the measurement values were converted into a graph against the concentrations of the standards. The quantity of the target substance in the sample was determined by employing this calibration curve. The measurement values and calibration curve are demonstrated in Figure S3.

Similarly, a calibration study was performed for ESC. A series of standard solutions were prepared using an ESC stock solution (1000 mg·L^–1^). The quantitative analysis of ESC was carried out using a UPLC-DAD system at a detection wavelength of 240 nm. The calibration curve was constructed by plotting peak areas against the corresponding concentrations and exhibited a high degree of linearity over the investigated concentration range. Using this calibration curve, the amount of the target compound in the samples was determined. The calibration data and the resulting calibration curve for ESC are presented in Figure S4.

#### 3.3.2 Effect of Sample Solution pH on the Adsorption-Based Removal of MB and ESC

The relationship between adsorption efficiency and the pH of the sample solution has a significant effect on both the surface properties of the adsorbent and the chemical structure of the analytes. Based on the pH optimization studies (Figure 4A,E), the highest removal efficiencies for both MB and ESC were achieved at pH 6.0, which was subsequently selected as the optimum pH for further experiments. To provide a mechanistic explanation for these results, zeta potential analysis was performed to identify the surface charge characteristics of the Nanoclay@VS_2_ NFs (Figure 4B). The point of zero charge (pHpzc) was determined to be approximately 2.0. The surface potential transitioned from +0.19 mV at pH 2.0 to a significant negative value of −21.1 mV at pH 3.0, confirming that the adsorbent surface is predominantly negatively charged at pH values above 2.0. This negative charge density further increases as the pH moves toward 6.0, primarily due to the deprotonation of silanol groups in the nanoclay and the presence of sulfur-rich active sites in the VS_2_ structure.^43^ At the optimum pH of 6.0, the Nanoclay@VS_2_ NFs possess a robust negative surface charge. Under these conditions, MB (positively charged due to its p*K*_a_ of 3.8) and ESC (positively charged at pH 6.0 due to its p*K*_a_ of ∼9.5) exhibit strong electrostatic attraction toward the negatively charged surface of the nanocomposite.^44–46^

**Figure 4:**
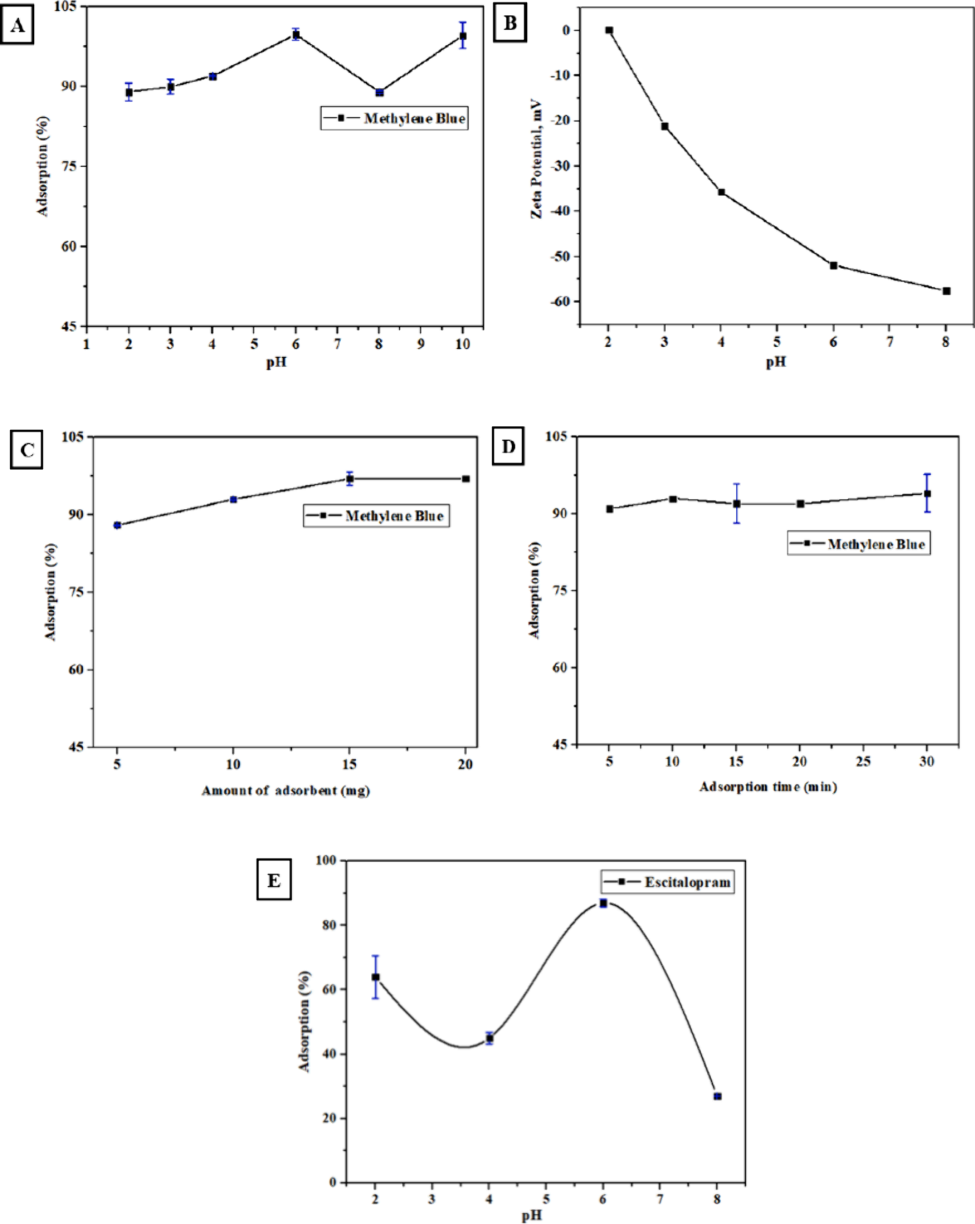
(A) Effect of sample solution pH on the adsorption efficiency of MB, (B) zeta potential measurement results for Nanoclay@VS_2_ NFs, (C) effect of Nanoclay@VS_2_ NF amount on the adsorption efficiency of MB, (D) effect of adsorption time on the adsorption efficiency of MB, and (E) effect of sample solution pH on the adsorption efficiency of ESC (*N* = 3).

Beyond electrostatic forces, the adsorption mechanism is further enhanced by nonelectrostatic interactions. The 2D layered architecture of VS_2_ nanoflowers facilitates π-π stacking interactions with the aromatic rings of MB and ESC. Additionally, the presence of hydroxyl groups on the nanoclay surface allows for the formation of hydrogen bonds with the heteroatoms (N, O) of the pollutants. The hierarchical nanoflower morphology also prevents the restacking of clay layers, maintaining accessible active sites and contributing to pore-filling effects. These findings demonstrate that the pH-dependent removal performance is a synergistic result of electrostatic attraction, aromatic stacking, and hydrogen bonding, as confirmed by the zeta potential and p*K*_a_ correlations.^43–47^

#### 3.3.3 Effect of Adsorbent Amount on the Adsorption-Based Removal of MB

The effect of the amount of Nanoclay@VS_2_ NFs on adsorption efficiency was investigated using 5–20 mg (Figure 4C). The findings indicated that an increasing in the quantity of adsorbent resulted in a concomitant rise in adsorption efficiency. It was observed that maximum adsorption efficiency was achieved with the addition of 15 mg of Nanoclay@VS_2_ NFs. Consequently, 15 mg of Nanoclay@VS_2_ NFs was utilized in the ensuing phases of the adsorption technique.

#### 3.3.4 Effect of Adsorption Time on the Adsorption-Based Removal of MB

The adsorption time is a pivotal parameter that exerts a direct influence on the efficiency and effectiveness of the adsorption process. Optimising the adsorption time is a critical step in increasing the efficiency of the process. In order to ascertain the optimum adsorption time, model solutions were subjected to mixing with Nanoclay@VS_2_ NFs for periods ranging from 5 to 30 min. The optimum time was determined to be 10 min (Figure 4D).

#### 3.3.5 Effect of Initial Pollutant Concentration on Adsorption Capacity and Removal Efficiency

Adsorption capacity is a pivotal property that signifies the efficacy with which an adsorbent can adsorb a particular analyte. Adsorption capacity is defined as a quantitative measure of the amount of analyte adsorbed per unit of adsorbent. In this study, the adsorption capacity of Nanoclay@VS_2_ NFs for MB was investigated. The adsorption method was applied in a 40 mL model solution containing increasing MB concentrations (5, 10, 20, 40, 80, and 100 mg·L^–1^). Fifteen mg of adsorbent was used. Subsequent to the adsorption process, the samples were measured at a wavelength of 664 nm, utilizing a UV–vis spectrophotometer. The quantity of analyte adsorbed per adsorbent was determined by calculating the obtained absorbance values. The equilibrium adsorption capacity (Qe) of the nanomaterial was calculated using eq 2:^23^

$$Q_{e}=\frac{C_{0-C_{e}}}{m}\times V$$ 7

In this equation, Q_e_ (mg·g^–1^) indicates the equilibrium adsorption capacity, C_o_ (mg·L^–1^) denotes the initial concentration, C_e_ (mg·L^–1^) indicates the equilibrium concentration, V (L) indicates the volume of the solution, and m (g) indicates the amount of adsorbent.^24^ Consequently, the equilibrium adsorption capacity and percentage graph of MB are presented in Figure 5.

**Figure 5:**
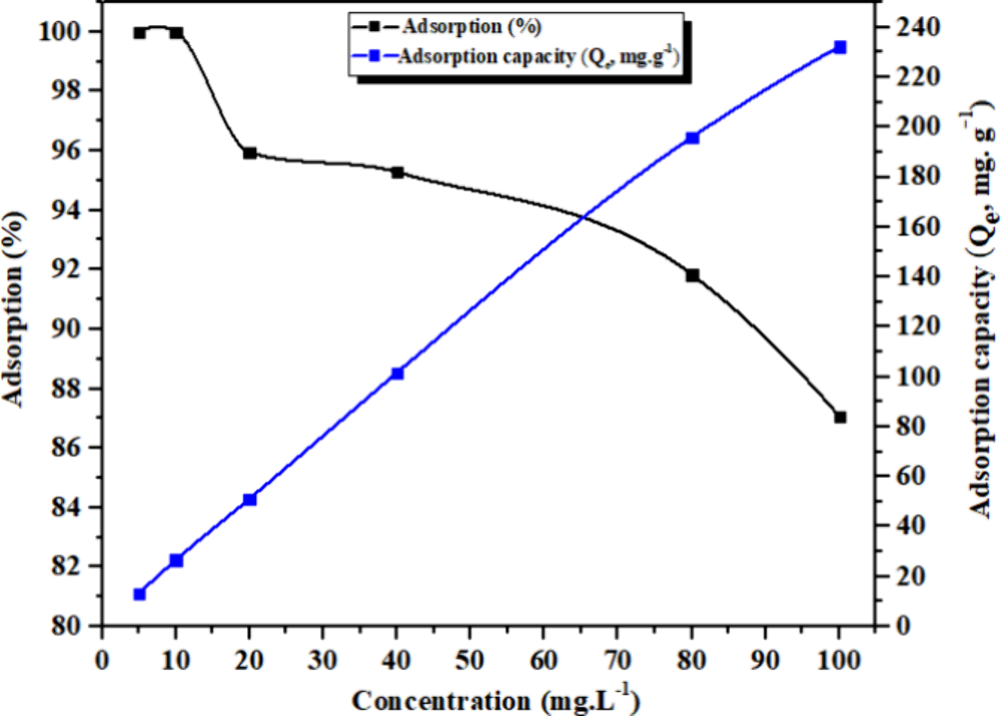
Effect of initial pollutant concentration on the adsorption capacity (qe) and removal efficiency (H%) of MB using Nanoclay@VS_2_ NFs (*N* = 3).

The effect of initial concentration on the adsorption performance is illustrated in Figure 5. As the initial MB concentration increased from 5 to 100 mg·L^–1^, the adsorption capacity (q_e_) exhibited a significant upward trend, reaching a maximum value of 232.19 mg.g^–1^. This observation can be explained by the fact that the increase in the number of dye molecules in the solution enhances the probability of interactions with the active sites on the adsorbent surface, thereby leading to an increase in the amount of substance adsorbed per unit mass. Conversely, the removal efficiency (%) showed a gradual decline from 100% to 87.07% at higher concentrations. This decrease is attributed to the limited availability of active sites relative to the increasing number of dye molecules, resulting in the progressive saturation of the adsorbent surface.

#### 3.3.6 Effect of Temperature on the Adsorption-Based Removal of MB

To investigate the effect of temperature on adsorption, experiments were conducted using 15 mg of Nanoclay@VS_2_ NFs adsorbent, 30 mg·L^–1^ MB solution, 1.5 mL of pH 6.0 buffer solution, and a sample volume of 40 mL. Samples were subjected to different temperature conditions (20, 30, 40, 50, and 60 °C) were processed simultaneously mixing. During the experiment, sample collection times were set at 10, 20, 30, 40, 50, and 60 min, and samples collected at the specified times for each temperature were transferred to Eppendorf tubes. The collected samples were analyzed using a UV–vis spectrophotometer. The adsorption efficiencies obtained are presented in Table 1. In this study, no significant change was observed in the adsorption of MB at different temperatures, with removal efficiencies remaining consistently high (82%–96%) across the tested range. The preservation of removal efficiency despite the increase in temperature confirms that the Nanoclay@VS_2_ NFs possess high thermal stability and a robust morphology. This observation indicates that the adsorption process is not limited to weak physical forces; rather, it is governed by a synergy between electrostatic attraction and more stable interactions, such as π-π stacking and hydrogen bonding. This multidimensional interaction network is the fundamental factor enabling the synthesized nanocomposite to maintain its structural integrity and exhibit high performance even under varying environmental conditions.

**Table 1:** Adsorption Efficiency of MB Solution at Specific Temperatures and Contact Times

adsorption (%)					
**time (min**)	20 °C	30 °C	40 °C	50 °C	60 °C
10	85	82	95	89	83
20	90	89	93	94	87
30	94	89	95	89	92
40	89	85	94	88	91
50	93	87	94	86	95
60	96	87	96	93	94

The results show that the produced Nanoclay@VS_2_ NFs adsorbent can be applied to the removal of MB in solutions over a wide temperature range. This shows that this developed method can be directly used in the removal of wastewater from industrial facilities with wastewater at different temperatures.

#### 3.3.7 Investigation of Adsorption Isotherms

The relationship between adsorbent surface properties and the amount of contaminant in solution is explained by isotherm models. The Langmuir isotherm is predicated on the assumption that adsorption occurs in a single layer on homogeneous surfaces. Conversely, the Freundlich isotherm posits the notion that adsorption transpires across multiple layers on heterogeneous surfaces.^48^ The adsorption process between MB and Nanoclay@VS_2_ NFs was modeled using the Langmuir and Freundlich isotherm models. The graphs drawn for the Langmuir and Freundlich isotherm models related to the adsorption of MB onto Nanoclay@VS_2_ NFs are presented in Figures 6A,B, respectively. The Langmuir isotherm R^2^ values for Nanoclay@VS_2_ NFs were found to be 0.939, and the Freundlich isotherm R^2^ value was 0.9997, indicating that the adsorption process is consistent with the Freundlich isotherm model. This finding suggests that the adsorption process occurs on a heterogeneous surface, where the adsorption sites possess unequal energy levels.

**Figure 6:**
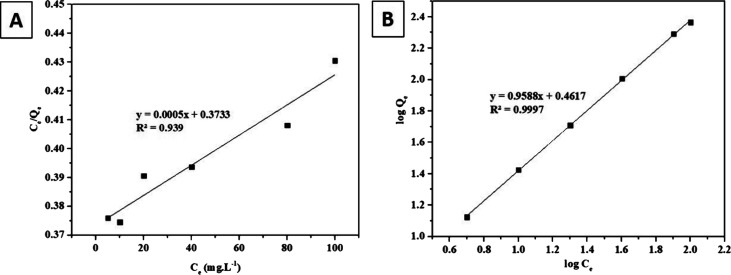
(A) Langmuir isotherm graph of Nanoclay@VS_2_ NFs; (B) Freundlich isotherm graph of Nanoclay@VS_2_ NFs.

### 3.4 Photocatalytic Removal of MB and ESC with Nanoclay@VS_2_ NF photocatalyst

A model solution containing MB was prepared for the purpose of determining the performance of the Nanoclay@VS_2_ NF photocatalyst in photocatalytic degradation experiments. A UV-halogen lamp was placed in a quartz sleeve at the center of the photocatalytic reactor. The volume of the model solution exposed to UV light was set at 150 mL, and the prepared model solution was placed approximately 5–7 cm below the UV-halogen lamp inside the photocatalytic reactor and continuously stirred with a magnetic stirrer. Prior to UV illumination, the suspension was kept in a dark environment for a specific period (12 h for MB and until equilibrium for ESC) to achieve adsorption–desorption equilibrium. This dark phase is crucial to differentiate the removal achieved by physical adsorption from the subsequent photocatalytic degradation. By reaching this equilibrium, the initial concentration for the photocatalytic process was established, ensuring that the observed decline in pollutant concentration under UV light is directly attributed to the catalytic activity of the Nanoclay@VS_2_ NFs.

For the model solution, 30 mL of buffer solution was first added to MB at a concentration of 93 mg·L^–1^, and the final volume was adjusted to 150 mL. 100 mg of Nanoclay@VS_2_ NFs was added to the prepared 150 mL model solution, and the mixture was left in a dark environment until the adsorption of the organic molecule was complete. The mixture was then transferred to a photocatalytic reactor and exposed to a UV-halogen lamp. The duration under the UV lamp was set to 480 min. To determine the photocatalytic degradation rate over time, approximately 2.0 mL samples were taken at specified time intervals (30 min/sample) and transferred to Eppendorf tubes for analysis. Measurements were performed at a wavelength of 664 nm using a UV–vis spectrophotometer. Figure 7B shows the changes in the absorbance spectrum of MB as a function of wavelength due to photocatalytic degradation on the Nanoclay@VS_2_ photocatalyst, while Figure 7C illustrates the degradation of MB as an effect of the exposure time to UV light on the Nanoclay@VS_2_ NF photocatalyst. At the same time, the decrease in absorbance value also indicated the degradation of MB. In the study conducted using Nanoclay@VS_2_ NFs, the MB degradation rate was found to be 90%.

**Figure 7:**
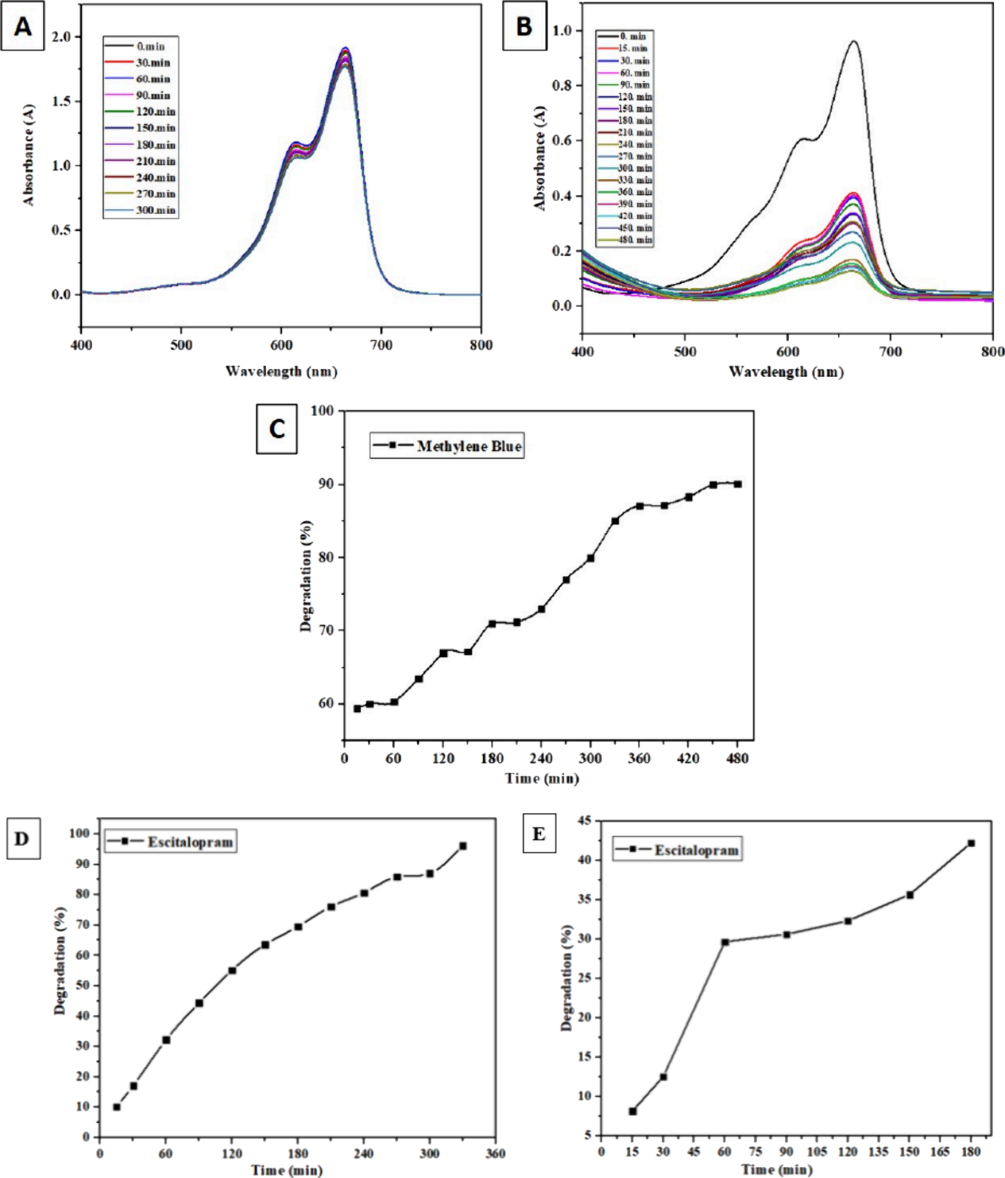
(A) Changes in the absorbance spectrum of MB degradation over time in an environment without a photocatalyst, (B) changes in the absorbance spectrum of MB with photocatalytic degradation on the Nanoclay@VS_2_ NF photocatalyst as a function of wavelength, (C) degradation of MB by the Nanoclay@VS_2_ NF photocatalyst as a function of UV exposure time, (D) degradation of ESC by the Nanoclay@VS_2_ NF photocatalyst as a function of UV exposure time, and (E) degradation of ESC as a function of UV irradiation time in the absence of a photocatalyst.

The photocatalytic degradation exchange of ESC was investigated under the same standard conditions. For the model solution, 30 mL of buffer solution was added to ESC at a concentration of 10 mg·L^–1^, and the final volume was adjusted to 150 mL. 100 mg of Nanoclay@VS_2_ NFs was added to the prepared 150 mL model solution. To reach adsorption equilibria, the mixture was stirred in a dark environment and then exposed to UV light for 330 min. The degradation process was monitored from predetermined intervals of 2.0 mL samples; these samples were filtered and analyzed using a UPLC-DAD system. The ESC degradation rate determined by Nanoclay@VS_2_ NFs was found to be 96% Figure 7D.

A blank sample analysis was performed by the addition of 150 mL of distilled water to the MB solution, resulting in a concentration of 10 mg per liter. As illustrated in Figure 7A, the degradation of MB is evident following exposure to UV light in the absence of a photocatalyst. The data obtained from this experiment indicate that a 5% degradation occurred after 300 min. The findings indicate that the degradation efficiency of MB is remarkably low in the absence of a photocatalyst.

The blank sample analysis for ESC revealed a 42% degradation rate under the same experimental conditions. This result suggests that ESC is more susceptible to UV light and more prone to direct photodegradation compared to MB Figure 7E.

The results demonstrated that the degradation efficiency of organic pollutants remained limited in the absence of a photocatalyst. This situation clearly indicates that the use of a photocatalyst plays a pivotal role in the degradation processes and serves as a decisive factor in enhancing efficiency for both MB and ESC, particularly at higher concentrations.

The photocatalytic degradation process conducted with the Nanoclay@VS_2_ NFs nanocomposite is based on the fundamental operating principles of semiconductor photocatalysis. In this mechanism, when the photocatalyst is excited by light of appropriate energy, electrons in the valence band transition to the conduction band, generating electron–hole (e^–^/h^+^) pairs. These photogenerated charge carriers trigger various redox reactions on the surface: electrons in the conduction band react with dissolved oxygen to form superoxide radicals (O_2_^–^), while holes in the valence band interact with water or hydroxide ions to produce hydroxyl radicals (·OH).^51^ The photocatalytic efficiency was evaluated under a 400 W UV-halogen lamp, which provides a broad continuous emission spectrum. Although the Nanoclay@VS_2_ composite exhibits a direct band gap of 3.77 eV, the high intensity irradiation provided by the 400 W source in the UV region ensures a sufficient photon flux in the 300–330 nm range for the electronic excitation of the catalyst. Furthermore, the presence of structural defects such as sulfur vacancies in the VS_2_ lattice and sub-bandgap transitions often associated with the Urbach tail effectively extends the light-harvesting capability slightly beyond the fundamental absorption edge (e.g., 340–360 nm). These structural disorders create intermediate energy levels or midgap states below the conduction band, facilitating effective photocatalytic performance under broad-spectrum illumination by utilizing the high photon density at the lower spectral limit.^52,53,62^ These reactive oxygen species (ROS), which possess potent oxidizing properties, play a critical role in the complete mineralization of complex organic pollutants such as MB and ESC.^51^ At this stage, the coexistence of Nanoclay and VS_2_ architectures facilitates the separation of charge carriers and reduces the recombination rate, thereby significantly enhancing the overall photocatalytic efficiency. In this context, UV radiation was specifically preferred to observe this mechanism at maximum efficiency and to evaluate the full potential of the Nanoclay@VS_2_ NFs composite. Theoretically, for the initiation of the photocatalytic process, the photon energy (hν) must be equal to or greater than the bandgap energy (E_g_) of the semiconductor. High-energy UV light, compared to visible light, maximizes the formation rate of electron–hole pairs and, consequently, the production of reactive radicals. While the visible-light activity of VS_2_ based materials is well-documented in the literature, UV radiation was selected as the primary model in this study to clearly observe the synergistic relationship between adsorption and photocatalysis under maximum quantum yield conditions.

Consistent with the 42% direct photolysis observed in the control experiments, the introduction of the Nanoclay@VS_2_ photocatalyst significantly enhanced the removal efficiency to 96%. This substantial improvement, which represents a net catalytic contribution of 54% over the photolytic background, indicates a strong synergistic effect between the catalyst surface and UV irradiation. Nanoclay@VS_2_ provides additional active sites for the generation of potent reactive oxygen species (ROS), which effectively facilitate the degradation of stable intermediate products that are otherwise resistant to direct photolysis.^54–56^ This enhanced performance confirms that while UV light initiates the transformation, the presence of Nanoclay@VS_2_ is the decisive factor for achieving near-complete degradation.

Consequently, the photocatalytic performance of the Nanoclay@VS_2_ is clearly distinguished from the inherent photolytic pathway, demonstrating its superior efficiency in achieving rapid and comprehensive pollutant remediation.

The photocatalytic degradation of MB and ESC was confirmed by the simultaneous disappearance of their characteristic UV–vis absorbance peaks and the significant decline in UPLC peak intensities. These results demonstrate that the Nanoclay@VS_2_ system effectively targets and cleaves the stable aromatic frameworks and functional groups of the pollutants. According to the literature, such degradation of the parent molecular structure is a primary indicator of photocatalytic success, as it marks the elimination of the molecule’s original chemical identity and its associated environmental persistence.^34^ The radicals generated in the Nanoclay@VS_2_ system ensure a rapid structural transformation, breaking down the molecules into smaller intermediate products. Therefore, this integrated analytical approach, combining both optical and chromatographic techniques, provides a robust validation of the material’s high photocatalytic performance and environmental suitability in remediating complex organic contaminants.

Furthermore, a significant temporal difference was observed between the adsorption and photocatalytic processes. While the adsorption of MB reached equilibrium rapidly within 10 min, the ESC removal was evaluated at its optimized equilibrium state to ensure maximum interaction. In contrast, the photocatalytic degradation required significantly longer durations to achieve high removal efficiencies, specifically 270–480 min for MB and 330 min for ESC. This discrepancy is primarily attributed to their distinct operational mechanisms. Adsorption is a rapid surface phenomenon driven by immediate electrostatic interactions and physical sequestration of the molecules onto the high-surface-area Nanoclay@VS_2_ NFs. Conversely, photocatalysis is a complex, multistep chemical reaction involving the generation of reactive oxygen species (ROS) that systematically break down molecular bonds. While adsorption effectively transfers the pollutants from the aqueous phase to the catalyst surface, the photocatalytic process is responsible for the chemical degradation and mineralization of these molecules, which inherently requires a longer duration to achieve near complete remediation.

The contribution of the photocatalytic process is essential for the overall efficiency of the Nanoclay@VS_2_ system beyond simple phase transfer. While a significant portion of the pollutants is initially removed through adsorption, the subsequent photocatalysis targets the molecules accumulated on the catalyst surface. This degradation prevents the saturation of the active sites on the Nanoclay@VS2 NFs and facilitates the continuous ‘regeneration’ of the surface. Thus, the synergistic relationship between adsorption and photocatalysis ensures that the material does not merely trap pollutants but actively mineralizes them, leading to a more comprehensive remediation compared to using adsorption alone.

#### 3.4.1 Effect of Solution pH on Photocatalytic Removal of MB and ESC with Nanoclay@VS_2_ NF Photocatalyst

The efficiency of photocatalytic processes is contingent upon the pH of the reaction solution.^49^ The pH of the solution can affect the surface charge of the photocatalyst, and consequently its photocatalytic performance.^50^ In order to evaluate the effect of solution pH on photocatalytic degradation, a series of photocatalytic experiments were conducted at a range of pH values (2.0–8.0) using MB dye and Nanoclay@VS_2_ NF photocatalyst (Figure 8A). The highest photocatalytic degradation efficiencies of the Nanoclay@VS_2_ photocatalyst were obtained at pH 4.0, and it was observed that degradation ratio of MB reached to %90 within 330 min. In addition, photocatalytic experiments were conducted at pH values (2.0–10.0) to evaluate the effect of solution pH on photocatalytic degradation using Nanoclay@VS_2_ NF photocatalyst for ESC (Figure 8D). The highest photocatalytic degradation efficiency of Nanoclay@VS_2_ photocatalyst was obtained at pH 2.0, and it was observed that the degradation rate of ESC reached 96% within 330 min. Photocatalytic degradation occurs on the surface of photocatalyst. Consequently, an increase in the amount of adsorbed material on the surface results in an enhancement of the photocatalytic efficiency. In photocatalytic processes, the initial stage of the reaction is adsorption, and molecules adsorbed onto the surface have the potential to react on the catalyst surface. An enhancement in adsorption ensures that a greater quantity of material reaches the catalyst surface, thereby increasing the photocatalytic efficiency.

**Figure 8:**
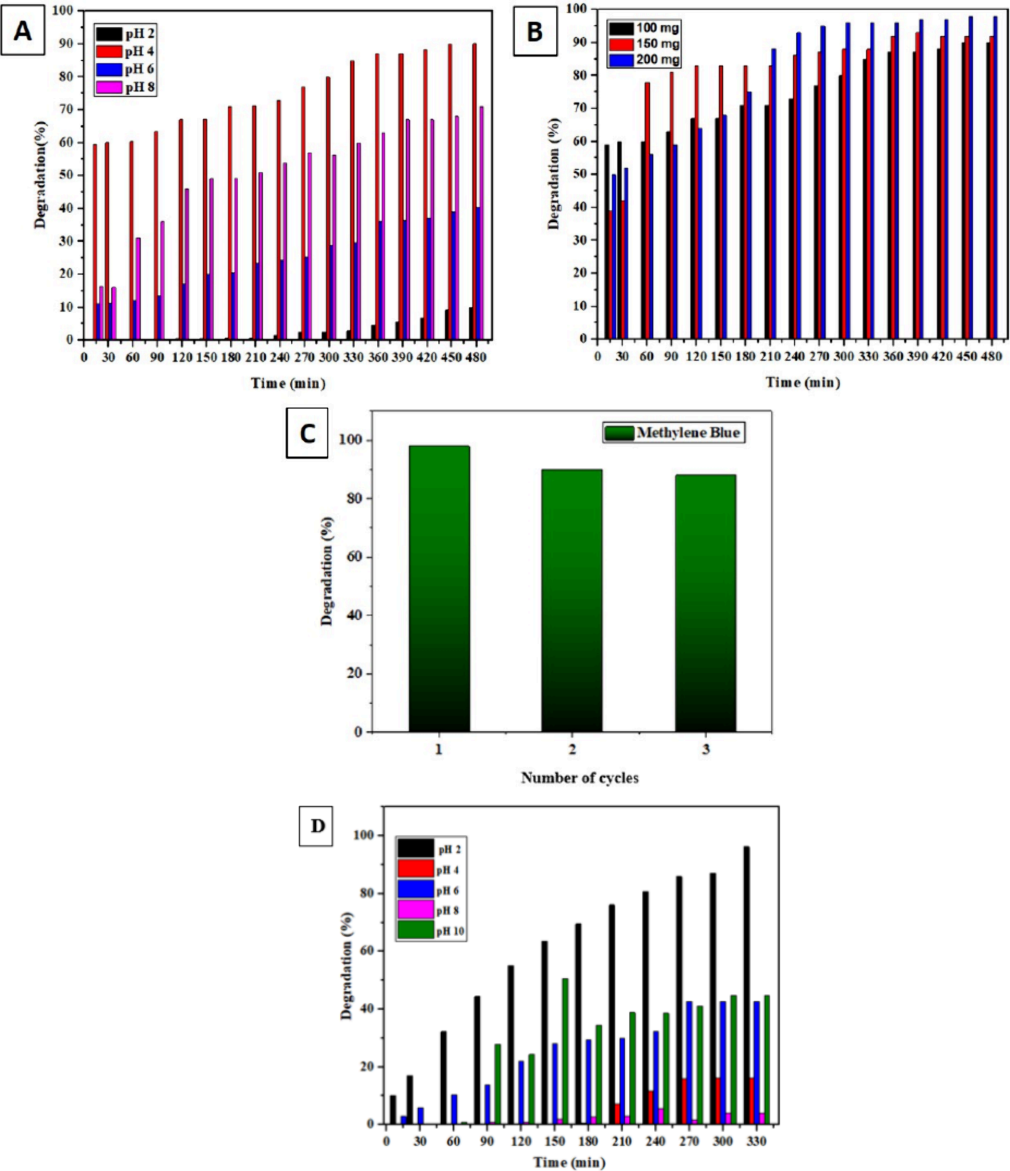
(A) Effect of solution pH on photocatalytic removal of MB with Nanoclay@VS_2_ NF photocatalyst, (B) effect of Nanoclay@VS_2_ NF photocatalyst amount on the photocatalytic degradation of MB and (C) effect of reusability of Nanoclay@VS_2_ NF photocatalyst on the photocatalytic degradation process of MB, and (D) effect of solution pH on the photocatalytic removal of ESC by Nanoclay@VS_2_ NF photocatalyst.

#### 3.4.2 Effect of the Amount of Nanoclay@VS_2_ NFs Photocatalytic Removal of MB

The effect of the amount of Nanoclay@VS_2_ NF photocatalyst on the photocatalytic degradation of MB was investigated (Figure 8B). Photocatalytic experiments were conducted using 100, 150, and 200 mg of Nanoclay@VS_2_ NFs to determine its effect on the photocatalytic degradation process. In the experiment with the addition of 100 mg of Nanoclay@VS_2_ NFs, a 90% yield was achieved in 480 min. Experiments conducted with the addition of 150 mg and 200 mg of Nanoclay@VS_2_ NFS achieved efficiencies of 90% and 95% at 390 and 270 min, respectively. The highest photocatalytic degradation efficiency was observed with 200 mg of Nanoclay@VS_2_ NFs. Therefore, Subsequent studies were continued with 200 mg of Nanoclay@VS_2_ NFs.

#### 3.4.3 Reusability of Nanoclay@VS_2_ NF photocatalyst

In photocatalytic reactions, the ability of photocatalysts to maintain their activity and to be reused while maintaining their performance is of great importance. When evaluated from an environmental and economic perspectives, the reusability of the Nanoclay@VS_2_ NF photocatalyst stands out as an important parameter. Therefore, the reusability of the Nanoclay@VS_2_ NF photocatalyst was tested under optimum conditions for photocatalytic degradation-based removal of MB. When the reusability cycle is examined in Figure 8C, while the photocatalytic degradation percentage of MB reached a very high value of 98% in the first cycle, this rate decreased to 90% in the second cycle and to 88% in the third cycle. This decrease shows that the photocatalytic activity of the photocatalyst is lost over time and its performance decreases, and the surface of the material may be saturated during this process.

#### 3.4.4 Photocatalytic Degradation Kinetics of MB

In the MB photocatalytic degradation study conducted using Nanoclay@VS_2_ NF photocatalyst, pseudozero, first-order, and second-order kinetic models were examined, and graphs representing these models are shown in Figure 9. Table 2 summarizes the rate constants k and their correlation coefficients (R^2^). When correlation coefficients (R^2^) were examined, the pseudozero-order model provided the best fit compared to the pseudo-first-order and pseudo-second-order models. Therefore, the removal rate of MB using the synthesized Nanoclay@VS_2_ NF photocatalyst was determined as a zero-order kinetic model (Figure 9A).

**Table 2:** Photocatalytic Degradation Kinetic Parameters of MB

	pseudo zero degree		pseudo first degree		pseudo second degree	
sample	*k*_0 _(mg·L^–1^_.min_^–1^_)_	R^2^	*k*_1 _(mg·L^–1^_.min_^–1^_)_	R^2^	*k*_2 _(mg·L^–1^_.min_^–1^_)_	R^2^
Nanoclay@VS_2_	0.0043	0.9879	0.0063	0.928	0.0103	0.81

**Figure 9:**
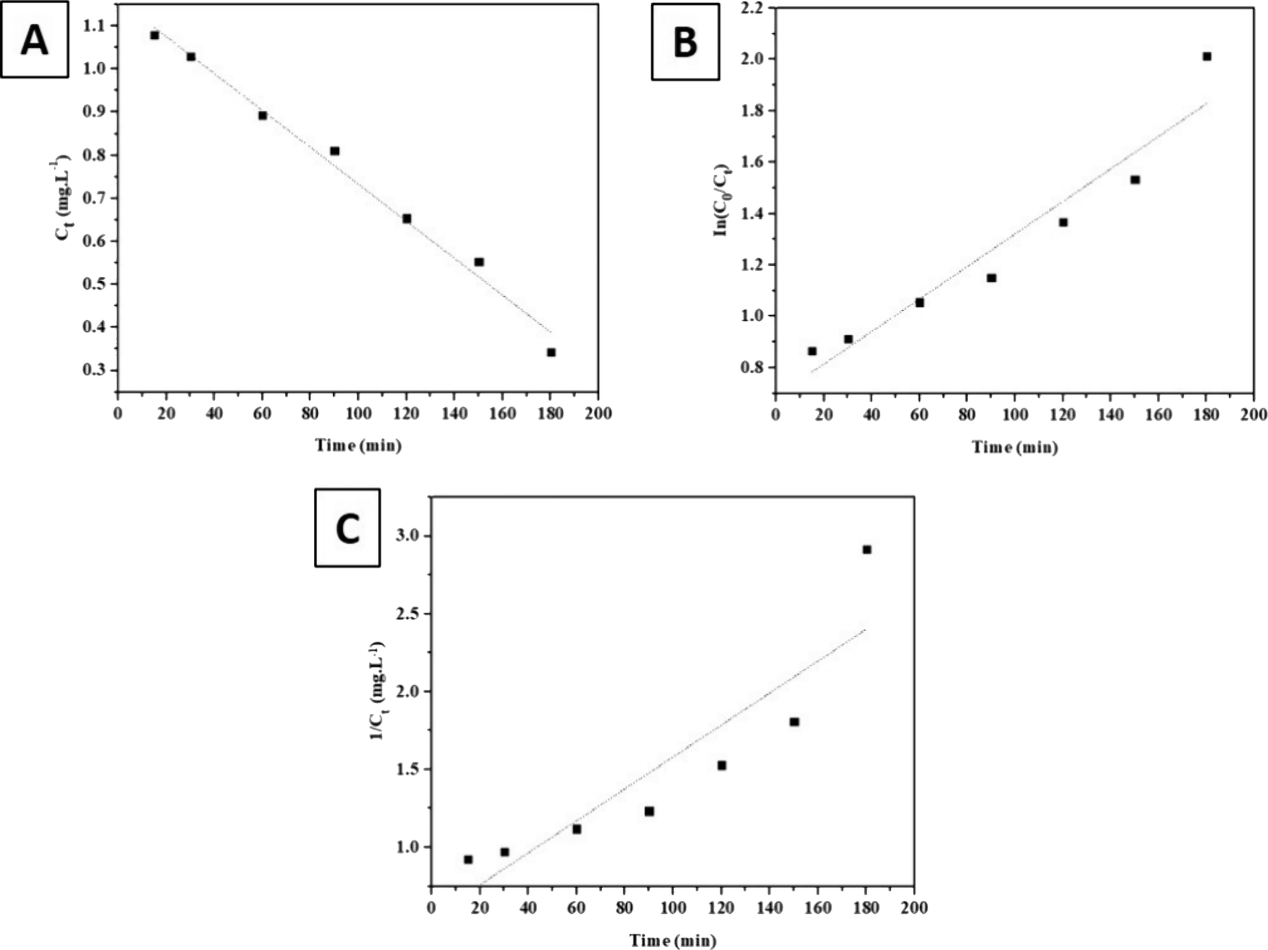
Kinetic plot for the photocatalytic degradation of MB. (A) Pseudozero-order model, (B) pseudo-first-order model, and (C) pseudo-second-order model.

The superior fit of the pseudozero-order model (R^2^ = 0.9879) over the traditional pseudo-first-order model indicates that, under the studied conditions, the degradation rate is independent of the bulk concentration of the pollutants. This kinetic behavior can be attributed to the high adsorption capacity of the nanoclay matrix, which leads to rapid saturation of the active VS_2_ sites (surface coverage, θ∼1) at the early stages of the reaction.^57^ Furthermore, the high optical density of the Nanoclay@VS_2_ composite may induce a screening effect, where light intensity-rather than pollutant concentration-becomes the limiting factor.^58^ The porous architecture of the nanoclay may also impose mass transfer limitations on the diffusion of pollutants to the internal active centers, shifting the process toward a diffusion-controlled zero-order regime.^59,60^ Consequently, the observed zero-order kinetics represent the surface-saturation limit of the Langmuir–Hinshelwood mechanism.

The degradation kinetics of MB and ESC were investigated to quantitatively evaluate the performance of the synthesized Nanoclay@VS_2_ composite. The experimental data were fitted to the zero-order kinetic model, and the apparent rate constants (kapp) were determined from the slope of the concentration–time plots. Furthermore, to determine the influence of the catalyst over direct photolysis, the Catalytic Enhancement Factor (EF) was calculated using the following eq 8:

$$\mathrm{EF}=\frac{\left(\mathrm{kapp},\mathrm{photocatalysis}\right)}{\left(\mathrm{kapp},\mathrm{photolysis}\right)}$$ 8

The calculated kinetic parameters are summarized in Table 3. For MB degradation, the kapp for the photocatalytic process (0.003518 min^–1^) was found to be significantly higher than that of direct photolysis (0.000185 min^–1^), resulting in a high EF value of 19.01. This indicates that the degradation of MB is almost entirely driven by the photocatalytic activity of Nanoclay@VS_2_. In the case of ESC, which is a more recalcitrant pharmaceutical molecule, a notable photolysis background (42% removal) was observed. However, the addition of Nanoclay@VS_2_ successfully accelerated the reaction kinetics, achieving an EF of 1.25. These quantitative results confirm that Nanoclay@VS_2_ effectively promotes the generation of reactive species, leading to enhanced degradation rates for both dye and pharmaceutical pollutants compared to UV exposure alone.

**Table 3:** Kinetic Parameters for the Removal of MB and ESC by Nanoclay@VS_2_ NFs

pollutant	system	time (min)	removal (%)	kapp (min^–1^)	EF
MB	photolysis (blank)	270	5	0.000185	
MB	Nanoclay@VS_2_	270	95	0.003518	19.01
ESC	photolysis (blank)	180	42	0.002333	
ESC	Nanoclay@VS_2_	300	87	0.002916	1.25

## 4 Conclusions

Multifunctional Nanoclay@VS_2_ NFs were synthesized and effectively used in the removal of MB and ESC, an organic pollutant, by adsorption and photocatalytic based removal methods. Nanoclay@VS_2_ NFs was a composite material consisting of the combination of nanoclay with high surface area and good adsorption capacity and vanadium disulfide (VS_2_) material with high photocatalytic property. This allows two important removal methods, adsorption and photocatalytic, to be performed simultaneously on a single material. A comparative evaluation of these two methods reveals that while adsorption is responsible for the rapid initial capture of pollutants, photocatalysis ensures their permanent chemical degradation. The Nanoclay@VS_2_ NFs composite performs best through the synergy of these two processes; high adsorption capacity facilitates the photocatalytic reaction by increasing the local pollutant concentration around the active sites, while photocatalysis prevents the saturation of the adsorbent by continuously breaking down the adsorbed molecules. This synergistic effect provides more effective removal efficiency in a shorter time. Furthermore, the hierarchical nanoflower (NF) morphology offers a significantly higher effective surface area compared to conventional nanoparticle structures. The 3D arrangement of the ‘petals’ within the nanoflower structure enhances light harvesting through multiple scattering and reflection, while simultaneously maximizing the exposure of catalytically active edge sites of VS_2_. This structural superiority facilitates a more robust synergy between the Nanoclay’s high adsorption capacity and VS_2_’s photocatalytic power, leading to performance levels that exceed those of single-phase materials. In the adsorption-based removal method, the optimum parameter conditions were determined as pH 6.0, the adsorbent amount as 15 mg and the adsorption time as 10 min, and the adsorption efficiencies varied between 93% and 99%. In the removal of MB by photocatalytic method, it was determined that MB was effectively decomposed with a decomposition efficiency of over 95% for 270–480 min under a 400 W UV lamp. As a result of photocatalytic experiments, optimum conditions were determined as pH 4 and 200 mg photocatalyst amount. In adsorption-based removal studies of ESC, the pH of the sample solution was set to 6.0, the amount of adsorbent to 15 mg, resulting in an adsorption efficiency of 87%. In photocatalytic experiments using pH 2.0 and 100 mg of Nanoclay@VS_2_ photocatalyst, it was shown that 96% of the ESC degraded within 330 min under a 400 W UV lamp. Reusability tests showed that the material maintained its effectiveness for three cycles. The findings obtained revealed that the developed photocatalytic system was quite successful and showed that the synthesized multifunctional nanocomposite has significant potential in terms of reusability. Literature studies using different techniques for the removal of MB were examined. Performance parameters of our study and other studies in the literature are given in Table 4. Better or similar performance was observed when compared to other methods in the literature.

**Table 4:** Comparison of Multifunctional Nanoclay@VS_2_ NFs Used as Adsorbent with Other Studies in the Literature^a^

nanomaterial	process	optimum pH	Initial MB/ESC concentration (mg·L^–1^)	adsorption capacity (mg g^–1^)	analyte	removal (%)	degradation (%)	source
CMMT	AD	9.5	-	158.79	MB organic pollutant			^61^
molybdenum disulfide (MoS_2_)	AD	6–12	20	200	MB organic pollutant	93.47		^62^
nanoclay	AD	8	500	338.15	MB organic pollutant	94.79		^63^
MMT@HTAB@PEG	AD	8–12	100	190.81–237.22	MB organic pollutant			^64^
Nanoclay@TiO_2_ @PNVP	AD + PC	10–10	20	3.3	MB organic pollutant	98	99	^24^
Nanoclay@VS_2_	AD + PC	6–4	10	232	MB organic pollutant	99	95	this study
Li_2_O-NPs	AD	8	10–50	46.86	ESC	>%90		^65^
TiO_2_ (P25)	PC	6.5	20		ESC		50	^66^
Nanoclay@VS_2_	AD + PC	6–2	10		ESC	87	96	this study

a Adsorption: AD; photocatalysis: PC.

These results show that Nanoclay@VS_2_ NFs can be used in the efficient removal of toxic organic pollutants such as MB and ESC from the water environment by exhibiting effective performance in both adsorption and photocatalytic processes. The integrated use of adsorption and photocatalytic degradation processes offers a potential solution in combating water pollution and constitutes an important alternative for advanced water treatment technologies. Future studies utilizing LC-MS/MS techniques could be conducted to identify degradation byproducts and clarify the comprehensive mineralization pathways of MB and ESC, thereby ensuring the environmental safety of the treated water.

## Supplementary Material



## Notes

Schematic representations of adsorption and photocatalytic degradation mechanisms (Figures S1 and S2) and calibration curves for MB and ESC (Figure S3) (PDF)
