# Supplementary material for: Use of Multifunctional Nanoclay@VS2 Nanoflowers in the Adsorption and Photocatalytic-Based Removal of Drug Molecules and Azo Dyes
Source: ACS Omega. 2026 Jun 2;11(23):33967–82. doi: 10.1021/acsomega.5c12735 (PMC13280915; doi:10.1021/acsomega.5c12735)
Supplement: Supplementary file 1 [file ao5c12735_si_001.pdf]

Use of Multifunctional Nanoclay@VS_2 Nanoflowers in the Adsorption and Photocatalytic-Based Removal of Drug Molecules and Azo Dyes

Fulya Sütçü Güney[1], Oktay Özkan[2], İbrahim Narin[3], Erkan Yılmaz[3,4,5,6]

[1]Erciyes University Faculty of Engineering, Institute of Science and Technology, Kayseri, Turkey

[2]*Environmental Engineering Department, Erciyes University, Kayseri, Turkey*

[3]Department of Analytical Chemistry, Faculty of Pharmacy, Erciyes University, 38280 Kayseri, Turkey

[4]Technology Research & Application Center (TAUM), Erciyes University, 38039 Kayseri, Turkey

[5]ERNAM-Nanotechnology Research and Application Center, Erciyes University, 38039 Kayseri, Turkey

[6]*Erciyes Teknopark-ChemicaMed Chemical Inc., Erciyes University Technology Development Zone, 38039 Kayseri, Turkey*

[*]Author of correspondence: erkanyilmaz@erciyes.edu.tr

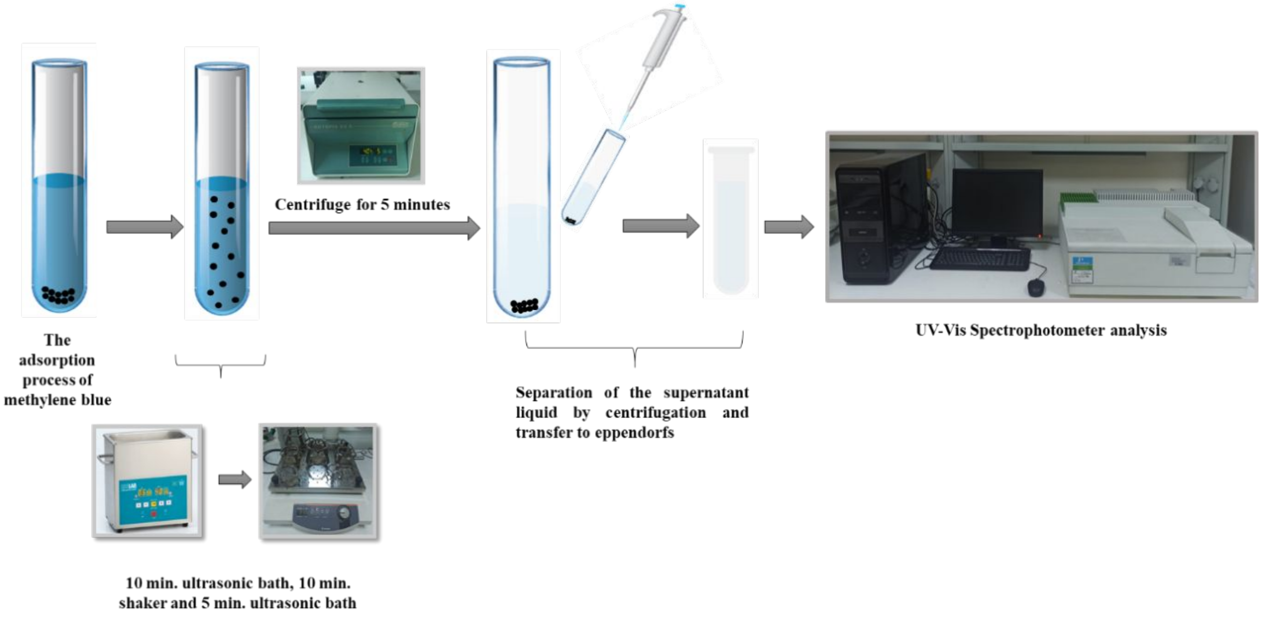

Figure S1. Schematic representation of the adsorption-based removal of MB by Nanoclay@VS$_2$ NFs.

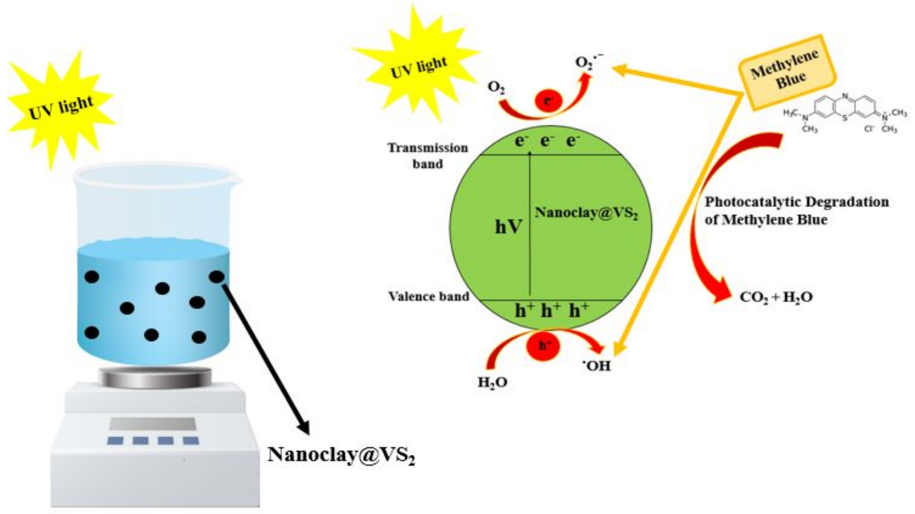

Figure S2. Schematic representation of the photocatalytic degradation-based - removal of MB by Nanoclay@VS$_2$ NFs.

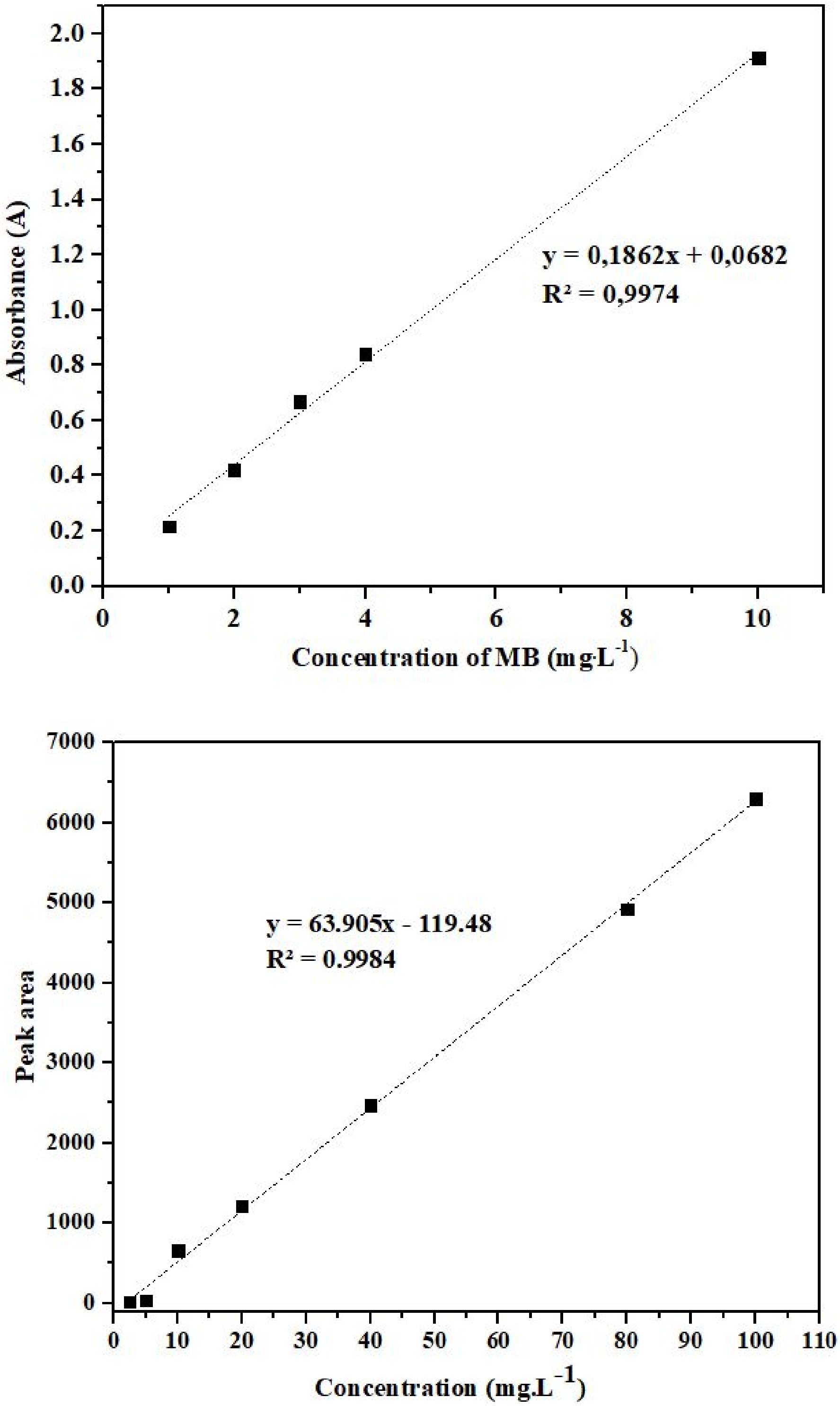

Figure S3. (A) Calibration curve graph for MB and **(B)** Calibration curve graph for ESC.
